# Community pharmacists’ views of using a screening tool to structure medicines use reviews for older people: findings from qualitative interviews

**DOI:** 10.1007/s11096-018-0659-z

**Published:** 2018-05-23

**Authors:** Karen Cardwell, Carmel M. Hughes, Cristín Ryan

**Affiliations:** 1 Department of General Practice, Health Research Board Centre for Primary Care Research, Royal College of Surgeons in Ireland, Dublin 2, Ireland; 2 School of Pharmacy, Queen’s University Belfast, 97 Lisburn Road, Belfast, BT9 7BL UK; 3 School of Pharmacy and Pharmaceutical Science, Trinity College Dublin, The University of Dublin, College Green, Dublin 2, Ireland

**Keywords:** Community pharmacists, Medicines use reviews, Older people, Prescribing appropriateness, Screening tools, Theoretical Domains Framework

## Abstract

*Background* The Medicines use review (MUR) service, provided by community pharmacists, seeks to optimise patients’ use of medicines. There is limited evidence on the clinical effectiveness of this service. Structuring MURs to include an assessment of prescribing appropriateness, facilitated by a validated prescribing screening tool, has the capacity to enhance this service. *Objective* To explore community pharmacists’ views on the facilitators and barriers towards the utilisation of a screening tool as a guide to conducting structured MURs. *Setting* Community Pharmacy, Northern Ireland. *Method* Using the 14 domain Theoretical Domains Framework (TDF), semi-structured interviews were conducted with community pharmacists. Interviews were digitally recorded, transcribed verbatim and analysed using the Framework method. *Main Outcome Measure* Pharmacists’ views towards utilisation of a screening tool as a guide to conducting structured MURs. *Results* Based on the analysis of 15 interviews, 11 TDF domains (‘Knowledge’, ‘Skills’, ‘Social and professional role and identity’, ‘Beliefs about capabilities’, ‘Beliefs about consequences’, ‘Reinforcement’, ‘Goals’, ‘Memory, attention and decision process’, ‘Environmental context and resources’, ‘Social influences’, ‘Behavioural regulation’) were deemed relevant. Facilitators included: knowledge of patients, clinical knowledge, perceived professional role, patients’ clinical outcomes, influence of peers. Barriers included: prioritisation of other clinical activities, inability to access patients’ clinical information, perceived alienation from the primary healthcare team and staffing issues. *Conclusions* Using the TDF, key facilitators and barriers were identified in the use of a screening tool as a guide to conducting MURs. These findings may assist in further development of MURs as a means to optimise patients’ medicines use.

**Electronic supplementary material:**

The online version of this article (10.1007/s11096-018-0659-z) contains supplementary material, which is available to authorized users.

## Impacts on Practice

- This study explored community pharmacists’ views on the facilitators and barriers towards the utilisation of a screening tool as a guide to conducting structured medicines use reviews (MURs).
- The main barriers identified were prioritisation of other clinical activities, inability to access patients’ clinical information, perceived alienation from the primary healthcare team, staffing issues and perceived professional identity.
- To overcome these barriers there is a need to improve communication and collaboration between GPs and community pharmacists, staffing issues need to be addressed and skills training in screening tool utilisation should be developed.
- This study provides preliminary data on the barriers and facilitators to utilising a screening tool as part of a MUR, which may assist in further development of MURs as a means to optimise patients’ medicines use.

## Introduction

Prescribing for older people is complex, due to the increased likelihood of developing multiple long-term conditions (multimorbidity) and prescribing of multiple medications (polypharmacy) [1]. Polypharmacy is an essential component in the management of multimorbidity, however it is associated with several undesirable outcomes such as reduced adherence to prescribed medication, drug–drug interactions and adverse drug reactions (ADRs) [1]; the latter is a particular concern in older people as the process of physiological ageing also renders this group more susceptible to ADRs [2]. Moreover, older people, particularly those with multiple long-term conditions, are routinely excluded from clinical drug trials and therefore the true safety and efficacy of many drugs has not been properly assessed in this population [3]. Consequently, the older age group are at an increased risk of potentially inappropriate prescribing (PIP), i.e. the under-, over- or mis-prescribing of medicines, which has been associated with ADRs, hospital admissions and mortality [4]. This has led to the development of a number of screening tools to evaluate the appropriateness of prescribing, e.g. Screening Tool of Older Persons’ Prescriptions (STOPP) and Screening Tool to Alert doctors to Right Treatment (START) [5, 6] and the Beers Criteria [7]. However, the potential of these tools to impact on long-term outcomes (e.g. mortality) has yet to be determined, since none is routinely used in clinical practice and there is a lack of robust data from randomised clinical trials [8].

One approach through which PIP can be identified and addressed is through an intervention such as a medication review. In an effort to improve prescribing and reduce polypharmacy, there has been an international drive to involve a range of healthcare professionals in medicines management. One aspect of this has been the implementation of a formal medication review by community pharmacists to identify issues of poor adherence and medication-related problems e.g. medication use where there is no indication, untreated indications and sub-therapeutic dosages [9]. This service is offered to patients throughout the world, the characteristics of which differ depending on the country in which it is being offered. In Australia, this service is called the Collaborative Home Medicine Review (HMR) service, Medication Therapy Management (MTM) service in the United States (US) [10] and Medicines use review (MUR) service in the United Kingdom (UK) [11].

In the UK, MURs are provided by accredited community pharmacists in the community pharmacy setting, in a suitable consultation area. The aim of a MUR is to improve patients’ adherence to therapy, ensure they are confident in using their medicines, resolve any problematic side-effects which may impede patients’ adherence to therapy and, in turn, improve the clinical and cost-effectiveness of prescribed therapy [11]. In England, Wales and Northern Ireland, the MUR service focuses on target patient groups (e.g. those with a particular chronic condition, or those taking high-risk medicines such as warfarin) and eligible patients are identified by the pharmacist. Following the review, completed MUR documentation, which may include written recommendations on the clinical and/or cost-effectiveness of the prescribed therapy, are forwarded to the patient’s GP [12]. Community pharmacists have been provided with general guidelines on how MURs should be undertaken, however there is no specific screening tool recommended to assist pharmacists in the medication review process [13]. To date, current evidence regarding the clinical and cost effectiveness of MURs has been inconclusive as there has been limited evidence reported on patients’ clinical outcomes [14]. Moreover, the quality of written reports provided to GPs and GPs’ hostility towards the service have been cited as further problems [15]. A small number of studies have explored pharmacists’, patients’ and GPs’ attitudes towards the MUR service. However, these results have been largely negative due to a variety of reasons such as patients’ perception that MURs serve no purpose [9, 12], pharmacists’ perception that GPs did not endorse MURs [16], as well as a lack of collaboration between GPs and community pharmacists [17].

The evident limitations of the MUR service present an opportunity to enhance this service [12]. For example, community pharmacists could undertake an assessment of prescribing appropriateness as part of a MUR, in an effort to improve prescribing, particularly in older people [9]. To facilitate this, MURs would need to be restructured to support community pharmacists in undertaking this assessment [18], e.g. through the application of a validated screening tool. To permit its use in the community pharmacy setting, the screening tool would be applied to medication data independent of clinical information and provide prescribing recommendations, to improve the appropriateness of prescribing. The enhancement of MURs (i.e. provision of a MUR to involve an assessment of prescribing appropriateness) was beyond the scope of this study. However, if this were to be developed, it would be important to identify the potential barriers and facilitators to including an assessment of prescribing appropriateness within a MUR.

### Aim of the study

The aim of this study was to establish community pharmacists’ views on the barriers and facilitators towards utilisation of a screening tool as a guide to conducting structured MURs, to improve the appropriateness of prescribing in older people.

### Ethics approval

Ethical approval was granted by the School of Pharmacy Ethics Committee, Queen’s University Belfast (QUB), on 26th February 2015 (Reference No. 011PMY2015).

## Method

The Theoretical Domains Framework (TDF) (12 domain [19] and 14 domain [20]) was developed by a group of experts as a means to help identify the factors (barriers and facilitators) to achieving behaviour change, particularly in the context of clinical practice. This study utilised the TDF to identify the barriers and facilitators towards utilisation of a screening tool as a guide to conducting structured MURs. Semi-structured interviews were conducted with community pharmacists using a TDF-based topic guide. This study was planned and reported in accordance with the ‘Consolidated criteria for reporting qualitative studies’ (COREQ) 32-item checklist [21].

### Sampling and recruitment strategy

Participants were purposefully sampled using a convenience sampling approach. Community pharmacists working in pharmacies affiliated with the Community Pharmacy Placement Network (which provides a structured placement programme for undergraduate Pharmacy students) at the School of Pharmacy (QUB), who had undertaken the necessary training in the provision of MURs, were eligible for inclusion in this study. At the time of this study, the Community Pharmacy Placement Network consisted of ~ 160 community pharmacies (which equated to ~ 32% of all community pharmacies in Northern Ireland). An alphabetical list of community pharmacists within the sampling frame was accessed by the researcher (KC). This list contained data relating to the name of the pharmacist, place of work, address, email address and work telephone number. The researcher (KC) telephoned each community pharmacist in the order they appeared on the list, to inform them of the study and explain why they had been selected as a potential participant. Thereafter, the researcher (KC) asked a screening question to confirm they had completed the necessary training in the provision of MURs. Community pharmacists, who expressed an interest in the study, were formally invited to participate in the study via an invitation letter, sent by email to the relevant community pharmacy, along with a study information sheet. Participants were given 1 week in which to decide whether they wished to participate. The anticipated sample size was approximately 20 participants. Recruitment continued until data saturation was reached, the identification of which was possible as data analysis was conducted in parallel to data collection. Participating pharmacists received an honorarium of £50 to cover their time committed to the study, (funded by The Harold and Marjorie Moss Charitable Trust), and were awarded a certificate of participation, following completion of the interview which could be used as evidence towards their Continuing Professional Development.

### Design of the interview schedule

Semi-structured interviews were utilised as the method of data collection for this study as they provide participants with an opportunity to develop and express their views [22] and allow the researcher to explore unexpected themes, whilst maintaining the structure and focus required to answer the research question [23]. To identify the barriers and facilitators which may influence changes to service provision, an interview schedule was developed. It comprised questions that were based on each of the domains contained within the TDF-14 domain version. The interview schedule included a clinical scenario to illustrate how a screening tool could be utilised as a guide to conducting MURs in the community pharmacy setting, and participants were provided with definitions for a MUR, PIP and screening tools (see Supplementary Data). Pilot interviews were conducted with four experienced pharmacist researchers (who are also experienced community pharmacists) acting as the interviewee and, subsequently, interview schedules were refined.

#### Data collection

Interviews were conducted face-to-face by the researcher (KC) in suitable locations (either participants’ place of work or the School of Pharmacy, QUB). Interviews were recorded using a digital recorder, with the participants’ written consent (obtained prior to the interview). Demographic data were also collected, to provide a description of the participating sample.

#### Data analysis

All participants were given a unique identifier to ensure confidentiality (e.g. Community Pharmacist 1-CP1). Data were analysed using the Framework method [24]. The TDF-14 domain served as the analytical framework, whereby each of the 14 domains served as a coding category. Data analysis consisted of the following steps:

*Transcription* Interview audio-recordings were transcribed verbatim (by the researcher [KC]) as soon as possible after the interview had taken place, which allowed the researcher (KC) to become familiar with the data [25]. Transcripts and audio recordings were stored on a password secure hard drive.

*Familiarisation* The familiarisation process involved repeated listening to the interview audio-recordings. All transcripts were checked for accuracy and imported into NVivo^®^ for analysis.

*Coding* Data were systematically coded by the researcher (KC) using a deductive approach, whereby each TDF-14 domain [20] served as a coding category. Three interview transcripts were randomly selected and were coded independently by two other members of the research team (CH, CR). The research team met to compare and agree upon the coding, and any coding discrepancies were resolved by consensus.

*Charting* The researcher (KC) charted (organised) the coded data into the framework matrix, which was generated as a Microsoft Excel^®^ spreadsheet [25].

*Content analysis* A content analysis of the framework matrix was then undertaken to identify subthemes (within each domain) relating to the barriers and facilitators to utilising a screening tool as a guide to conducting a MUR. The content analysis was completed using an inductive approach, as the subthemes that emerged during the content analysis were not pre-defined. Subthemes within each domain were summarised to give an overall impression of how each domain may influence the use of a screening tool as a guide to conducting MURs; this was illustrated using supporting quotes.

A summary of the content analysis was reviewed by two members of the research team (KC and CR) and a consensus on the relevance of each domain was achieved. A domain was regarded as ‘relevant’ if it was coded frequently within the transcripts and if the content of the domain was specifically related to the utilisation of a screening tool as a guide to conducting MURs. Transcripts were not returned to participants for comments and participants were not asked to provide feedback on the findings reported.

## Results

### Demographic overview of participating community pharmacists

In total, 18 pharmacists were recruited to take part in the study. Data saturation was deemed to have occurred at interview 15 as no new themes were identified by the researcher (KC) at this point. Therefore, three pharmacists who had previously been recruited for interview did not participate and no repeat interviews were necessary. Of those interviewed, nine (60.0%) were female and six were male (40.0%). The average length of time participants had been practising as pharmacists was 12.1 years (range 2–34 years). Table 1 details the demographic information relating to the community pharmacists enrolled in this study. The average duration of each interview was 39 min (range 24–72 min).

**Table 1:** Demographic data of community pharmacists

Pharmacist ID	Gender	Years practising as a pharmacist	Position	Multiple/independent pharmacy
CP1	Female	7	Pharmacy manager	Multiple
CP2	Female	2	Pharmacy manager	Multiple
CP3	Male	21	Pharmacy owner	Independent
CP4	Female	18	Pharmacy manager	Multiple
CP5	Female	11	Pharmacy manager	Independent
CP6	Male	34	Pharmacy owner	Independent
CP7	Male	8	Pharmacy manager	Multiple
CP8	Female	24	Pharmacy manager	Multiple
CP9	Female	4	Pharmacy manager	Independent
CP10	Female	2	Pharmacist	Independent
CP11	Male	20	Pharmacy manager	Multiple
CP12	Male	9	Pharmacy manager	Multiple
CP13	Female	12	Pharmacist	Independent
CP14	Female	5	Pharmacist	Independent
CP15	Male	5	Pharmacy manager	Multiple

*CP* community pharmacist

### Identification of barriers and facilitators perceived to influence enhancement of the MUR service

Domains regarded as relevant to the utilisation of a screening tool as a guide to conducting MURs were: ‘Knowledge’, ‘Skills’, ‘Social and professional role and identity’, ‘Beliefs about capabilities’, ‘Beliefs about consequences’, ‘Reinforcement’, ‘Goals’, ‘Memory, attention and decision process’, ‘Environmental context and resources’, ‘Social influences’ and ‘Behavioural regulation’. Those domains not regarded as relevant were: ‘Optimism’, ‘Intentions’ and ‘Emotions’. Table 2 provides a definition for each relevant TDF domain, reports the subthemes identified within each relevant domain and provides illustrative quotes.

**Table 2:** Definitions of the relevant TDF-domains, subthemes identified within each relevant domain and illustrative quotes

Definition of relevant TDF-domain	Subthemes within relevant TDF-domain	Illustrative quotes
Knowledge An awareness of the existence of something [23]	Clinical knowledge	“You would need up to date training on recent guidelines to make sure that your knowledge was up to date with any changes that have come in.” [CP4] “We have a very clear understanding of our patients, more so I do believe than GPs, because we see the same patients week after week. GPs now don’t really see the same patients. We have a really sound knowledge of their drug profiles…” [CP3]
	Knowledge of patients	
Skills An ability or proficiency acquired through practice [23]	Interpersonal skills	“…we can draw out the information from the patient first of all and then structure the rest of the MUR to fill in the gaps…” [CP11] “I think we need to be able to do it properly, we need workshops with live classes on how to conduct them properly…” [CP3]
	Skills to utilise a screening tool	
Social and professional role and identity A coherent set of behaviours and displayed personal qualities of an individual in a social or work setting [23]	Perception of their professional role	“We always try to look for instances where we can make a clinical difference…” [CP6] “They think it’s not your role as a pharmacist, that it’s their role as the doctor or even the asthma nurse.” [CP7]
	Perceived professional identity with others (GPs and other members of primary healthcare team, patients)	
Beliefs about capabilities Acceptance of the truth, reality or validity about an ability, talent or facility that a person can put to constructive use [23]	Confidence in their ability to utilise a screening tool	“…training on the use of the screening tool would be required…obviously I don’t understand how it would be used…” [CP11]
Beliefs about consequences Acceptance of the truth, reality or validity about outcomes of a behaviour in a given situation [23]	Consequences (both positive and negative) associated with utilising a screening tool	“…it potentially could save a lot of cash.” [CP13] “I think it would be good for then communicating that back to the like of the GP surgery, so that they can see that you’re following a structured guideline, rather than you’re just thinking that you know better than they do.” [CP4] “It might make them longer…it might add to the paperwork…” [CP14]
Reinforcement Increasing the probability of a response by arranging a dependent relationship, or contingency, between the response and a given stimulus [23]	Sense of making a clinical difference	“…to actually sit down with somebody for 20-30 min and work through any problems that they have, and to know that they have benefited from that, gives you job satisfaction.” [CP11] “If there was some sort of title that could be given or accreditation that would be favourable but it wouldn’t be the reason I would do it.” [CP5]
	Professional recognition	
Goals Mental representations of outcomes or end states that an individual wants to achieve [23]	Pharmacists’ goals	“…it would only be a priority after all the other things that I have to do get done…” [CP5] “As long as we got the go ahead with them [GPs] too.” [CP1]
Memory, attention and decision process The ability to retain information, focus selectively on aspects of the environment and choose between two or more alternatives [23]	MUR-focused strategies	“I think it would have to be easy to mark somewhere on the paperwork or the document online. You would need to have a carry through plan, so a point of action that sort of thing. But again, that would need to be prompted, that you had some action to take…” [CP8]
Environmental context and resources Any circumstance of a person’s situation or environment that discourages or encourages the development of skills and abilities, independence, social competence and adaptive behaviour [23]	Training resources and support	“…so it would be nice to have someone to go to or even someone to come in and train you on how to use it.” [CP2] “I don’t see how you could do it without clinical information…it just gently reminds you that you are doing the best to the best of your ability, but you are kind of working with your hands behind your back.” [CP6] “It is a struggle to get two MURs done a week for asthma or diabetes. There’s so many cut backs with staff…” [CP1] “We can’t do the initiative for nothing, which is what we are expected to do sometimes.” [CP3]
	Availability of patients’ clinical information	
	Staffing issues	
	Remuneration for services	
Social influences Those interpersonal processes that can cause individuals to change their thoughts, feelings or behaviours [23]	Influence of peers and professional bodies	“It’s got to be YOUR peers not a Dr XX from XXX University, he thinks it’s fantastic.” [CP6] “I think if you want us to provide more services, then doctors and nurses need to understand…I would want the doctors on board too.” [CP7] “…but I think if you ask some of the other members of the primary healthcare team, “Are you part of the primary healthcare team” they might disagree…I think it’s a grey area…it’s difficult to see how you are part of the primary healthcare team.” [CP6]
	Support of patients, GPs and other members of the primary healthcare team	
	Alienation of community pharmacists from other members of the primary healthcare team	
Behavioural regulation Anything aimed at managing or changing objectively observed or measured actions [23]	GP-focused strategies	“I probably would only see it if someone was coming back and being prescribed something again and that would then flash up. But otherwise I probably wouldn’t know if it had been changed or if the outcome had been achieved, unless the prescriber contacted me.” [CP15]

## Discussion

### Study synopsis

Using the TDF as the analytical framework, this study was an exploration of the barriers and facilitators, perceived by community pharmacists, towards the utilisation of a screening tool as a guide to conducting MURs. Based on the analysis of 15 interviews, the TDF-domains considered relevant to the application of a screening tool in a MUR were: ‘Knowledge’, ‘Skills’, ‘Social and professional role and identity’, ‘Beliefs about capabilities’, ‘Beliefs about consequences’, ‘Reinforcement’, ‘Goals’, ‘Memory, attention and decision process’, ‘Environmental context and resources’, ‘Social influences’ and ‘Behavioural regulation’. Restructuring and evaluating the MUR service would require a well-defined, robust methodological study, and was beyond the scope of this work. Nevertheless, this study serves as an exploration of the factors which would influence changes to the provision of the MUR service. These barriers and facilitators would need to be considered if the service were to be restructured. Subsequently, potential approaches to overcoming the barriers (or enhancing the facilitators) identified are described below.

### Potential approaches to overcoming the barriers (or enhancing the facilitators) identified

In an effort to overcome the barriers identified within the domains ‘Knowledge’, ‘Skills’ and ‘Beliefs about capabilities’, it is essential to provide education and training for pharmacists. These training sessions should target deficits in clinical knowledge, and describe the evidence base [26], which in this instance relates to the improvement of prescribing appropriateness, use of screening tools and the potential role of community pharmacists in improving prescribing appropriateness. To develop pharmacists’ skills relating to the operation of a screening tool in a MUR, it is important to provide skills training which should include: instruction on how to use a screening tool in a MUR as well as an opportunity for pharmacists to utilise it in a controlled setting (i.e. in a workshop, facilitated by those experienced in operation of the tool) [27]. It is anticipated that education and skills training would overcome pharmacists’ perceived lack of clinical knowledge (‘Knowledge) and skill (‘Skills’) and, in turn, increase pharmacists’ confidence in their ability (‘Beliefs about capabilities’) to conduct MURs using a screening tool designed to identify instances of PIP. Thereafter, the provision of continued training and support would enable pharmacists to develop further their professional practice (‘Environmental context and resources’). One approach to this could involve the use of ‘helpful others’, which has been advocated by Harding and Wilcock [28], whereby pharmacists are supported in their clinical practice through shared learning networks such as intranet, email groups and in-person meetings with other pharmacists.

Whilst education and training are paramount in overcoming the barriers associated with changing the provision of a service, they are not the only prerequisites. This has been evidenced in a study by Curran et al. [29] wherein education did not guarantee consistent application, by physicians, of a decision-making rule on the use of computed tomography. In this current study, although pharmacists had the desire to perform an increased number of MURs per week and believed the provision of MURs was part of their professional role, they recounted difficulty in achieving this due to restrictions on their time and limited availability of staff (‘Environmental context and resources’). This is consistent with previous research which has shown that pharmacists believe MURs are a valuable component of their professional role, but due to increasing work-loads and pressure from pharmacy managers to deliver on the quantity of MURs completed, pharmacists often undertake MURs with those patients on less complicated medication regimens. In turn, this prevents them from conducting a MUR with those patients who would benefit most and limits their opportunity to develop skills in providing MURs [28]. Thus, there is a need for pharmacy managers to address issues of inadequate staffing, to ensure that pharmacists have the appropriate level of support to allow them to fully embrace their clinical and professional role [17].

In this study, pharmacists supposed that GPs, other members of the primary healthcare team and patients questioned their professional role and clinical expertise regarding medication reviews and the assessment of prescribing appropriateness. Moreover, they reported the frustration of not being able to contact the GP directly. This resulted in them feeling isolated from the rest of the primary healthcare team (‘Social and professional role and identity’; ‘Social influences’). This is a significant barrier to the enhancement of the MUR service and is predominantly due to a lack of communication and collaboration between GPs and community pharmacists. This issue has been identified in previous studies and patients have also recognised that this has the potential to cause conflict between their GP and pharmacist, and impact on their own relationship with their GP [17, 30]. On the whole, poor communication and lack of collaboration between GPs and community pharmacists has been a longstanding and widespread issue in primary care [31–36]. This has led to the development of a number of theoretical models to understand factors associated with GP-community pharmacy collaboration [35].

In a recent qualitative study by Rathbone et al. [37] researchers identified a number of determinants of successful collaboration (between GPs and community pharmacists in Australia) to improve patients’ adherence to prescribed medicines. The barriers identified were a lack of shared location, a difference in perspectives, poor communication due to gatekeepers (GPs’ receptionists), irregular or volatile communication as well as a lack of mutual and professional respect. Conversely, the facilitators identified were access to a shared location, shared perspectives, face-to-face or electronic communication, regular communication as well as mutual and professional respect [37]. To overcome the barriers associated with ‘Social and professional role and identity’ and ‘Social influences’, collaboration between GPs and community pharmacists needs to improve. This could be facilitated through the provision of multidisciplinary educational programmes, organised between local GP surgeries and neighbouring pharmacies [32]. In addition, there needs to be a more focused effort to improve collaboration specific to the provision of MURs. Previous research has indicated the need to integrate clinical practice associated with the provision of MURs, in that MURs should be a shared partnership between community pharmacists and GPs, whereby patients are referred (by their GP) to their pharmacist for review [17]. As a result, GPs could be assured that the appropriate patients are being reviewed, pharmacists would have evidence that GPs approve of them conducting a medication review [27] and feelings of mutual and professional respect could be fostered [37].

In this instance, if the provision of MURs were to become a shared partnership between GPs and community pharmacists, ‘shared’ MUR documentation could be employed. Ideally, this documentation would be electronic and include reminders, in an effort to overcome the barriers identified with the domains ‘Memory, attention and decision process’ (e.g. cognitive overload, forgetting to address issues identified in the MUR) and ‘Social influences’ (inability to contact the GP directly). Hypothetically, if both parties are aware of the issues to be addressed in the MUR, both could be held accountable for any decisions made, or lack thereof. GPs would know which MURs needed to be followed-up (‘Behavioural regulation’) and they would not be over-burdened by endless (and perhaps irrelevant) MUR reports. As a result, both GPs and community pharmacists would have a vested interest in the MUR service, both would have shared perspectives and communication would be regular and proactive [37]. It is anticipated that, having overcome these barriers, community pharmacists may prioritise the provision of MURs (using a screening tool to identify instances of PIP) (‘Goals’), and positive consequences (perceived by pharmacists) such as an enhanced MUR service, improved clinical outcomes for the patient and reduced healthcare costs could be attained (‘Beliefs about consequences’).

This study used a systematic approach to identify the theoretical domains associated with the utilisation of a screening tool as a guide to conducting MURs, as a means to improving the appropriateness of prescribing in older people. The systematic approach used in this study was informed by recommendations made in previous TDF-based studies [38, 39]. The refined (TDF-14 domain version) TDF was used as the analytical framework [20]. This version has a strengthened empirical base as it was derived from the original TDF-12 domain version [19]. Data collected for this study were analysed independently and decisions were made through consensus approaches, which added to the reliability and validity of the results reported. Moreover, two members of the research team (CH, CR) have extensive experience in using the TDF as an analytical framework. Finally, community pharmacists recruited for this study were from a wide geographical area, working in independent pharmacies and pharmacy multiples, from both urban and rural settings. This should enhance the transferability of the findings from this study.

Despite the reported strengths of this study, there are also some limitations. Perhaps most obvious is the nature of the study. Since this is a qualitative analysis, the findings are not readily generalisable to the wider population. Whilst we appreciate that a convenience sampling approach has inherent limitations, this method was chosen due to time restrictions. The sampling frame comprised 160 pharmacies within the Community Pharmacy Placement Network, equating to almost a third of all community pharmacies in Northern Ireland. It may be the case that pharmacists working in these pharmacies may engage more frequently in pharmacy practice research which may have biased the results, however it should also be noted that all pharmacists are required to undertake 30 h of CPD annually, irrespective of whether their pharmacy is part of the Pharmacy Placement Network or not. Participants enrolled in the study were incentivised to take part. Subsequently, the study sample was self-selected, which can result in self-selection bias. This study is also limited in that the views of other key stakeholders (e.g. GPs and patients) were not explored. Had time permitted, this could have been undertaken as part of a larger study. Although this study discusses potential approaches to overcoming the barriers (and enhancing the facilitators) identified through the TDF domains, these approaches were not determined using the technique of mapping the domains to Behaviour change techniques (BCTs). The latter are considered to be the ‘active ingredients’ within an intervention [27]. Future research on this topic could follow this increasingly recognised methodology, leading to the development of a theoretically informed intervention [40]. Finally, prior to undertaking this piece of research, the researcher (KC) worked as a community pharmacist for a number of years. It was acknowledged that this could impart some bias to the data analysis process and subsequent findings reported in this study. However, in an effort to overcome this, a systematic approach to data analysis was undertaken and a consensus on the coding of transcripts and relevance of TDF domains was achieved between three researchers (KC, CH, CR).

## Conclusion

Using the TDF, this study explored the barriers and facilitators perceived (by community pharmacists) to influence the utilisation of a screening tool as a guide to conducting MURs, as means to improving the appropriateness of prescribing in older people. All domains (except ‘Optimism’, ‘Intentions’ and ‘Emotion’) were identified as relevant to changing the provision of the MUR service. These findings form the basis for considering how the barriers can be overcome and the facilitators enhanced.

## Electronic supplementary material

Below is the link to the electronic supplementary material.

Supplementary material 1 (DOCX 29 kb)
