# Supplementary material for: Community pharmacists’ views of using a screening tool to structure medicines use reviews for older people: findings from qualitative interviews
Source: Int J Clin Pharm. 2018 May 23;40(5):1086–95. doi: 10.1007/s11096-018-0659-z (PMC6208598; doi:10.1007/s11096-018-0659-z)
Supplement: Supplementary file 1 — Supplementary material 1 (DOCX 29 kb) [file 11096_2018_659_MOESM1_ESM.docx]

**INTERVIEW SCHEDULE FOR INTERVIEWS WITH COMMUNITY PHARMACISTS**

**Introduction**

“Hello, my name is Karen Cardwell and I am a researcher at Queen’s University Belfast. Thank you for taking the time to take part in this interview. This study is part of a PhD project which is examining the use of medicines in older people (i.e. those ≥65 years), with a particular focus on potentially inappropriate prescribing in this population.

The aim of this study is to establish your views on using a screening tool to conduct medicines use reviews (MURs), as a means of improving the appropriateness of prescribing for older people. During the interview, I hope to gather information regarding: the role of community pharmacists in the primary healthcare team; medicines management in older people; strengths and weaknesses of current MURs; and factors that may help or impede the use of a screening tool as a guide to conducting MURs.

I would like to remind you that the interview will be recorded to ensure we have an accurate and detailed account of what you say. I also want to check that you have had a chance to read through the information sheet that was sent out to you, and remind you that any information collected will be kept anonymous and will not be attributed to you. There are no right or wrong answers, so please be as honest as possible with your answers. You are free to stop the interview and/or recording at any time, and if there are any questions you prefer not to answer please let me know and we can move onto the next question.

Before we start, I need to get written consent from you. If you don’t mind, could you read through this consent form and initial each box to indicate that you understand and agree with each statement. (There are two copies, one for you to keep and the other I will keep for our records).

Have you any questions you would like to ask me before we start? Are you okay to proceed?”

*[Turn on digital recorder]*

**Demographic information**

1. Can you tell me what year you registered with the Pharmaceutical Society of Northern Ireland (PSNI) as a pharmacist?

2. How long have you been practising as a community pharmacist?

3. What is the title of your current post (e.g. pharmacy manager, locum pharmacist)?

4. And how long have you been in your current post?

5. Do you hold any additional qualifications, such as a Postgraduate Masters or non-medical prescribing certificate?

**Role of community pharmacists within the primary healthcare team**

6. Thinking specifically about the ‘primary healthcare team’, what is your understanding of this term?

*Prompts: Perhaps think of: other healthcare professionals (HCPs)/members of this team; HCPs you engage with on a daily basis.*

7. How do you view your role/responsibility within the primary healthcare team?

*Prompts: Perhaps think of factors that promote/hinder your role/responsibility within this team?*

**Community pharmacists’ engagement with older patients**

8. On a typical working day in your pharmacy, approximately how many older patients, or carers of older patients, would you encounter either face-to-face or over the telephone? When I say ‘older patients’, I am thinking of those over the age of 65 years.

9. Approximately what proportion of your overall dispensing activity is for older people?

10. Can you think of any potential problems associated with prescribing in older people?

*Prompts: For example: patients with a number of long-term conditions; patients taking a number of medicines; patients who experience an increased frequency/severity of side effects; patients living alone; patients who have problems managing their medicines.*

11. When dispensing prescriptions for older people, what common prescribing issues do you encounter?

*Prompts: Perhaps incorrect doses (excessive/sub-therapeutic doses); extended duration of therapy; inadequate management of side effects.*

**Role of community pharmacists in conducting MURs**

I now want to move on and talk about Medicines Use Reviews. Can I ask you to read this definition please?

*[Hand participant a card with the definition printed on it].*

**Medicines use review (MUR)**

The Business Services Organisation (BSO) defines a MUR as an appointment with a pharmacist to focus on how a patient is managing their medicines. The meeting is intended to: provide patients with information about the medicines they are taking; pick up any problems patients may be experiencing with their medicines; improve the effectiveness of a patient’s medicines; ensure medicines are appropriate for the patient.

12. To date, have you completed a MUR in a patient with asthma/diabetes?

13. In a typical working week, how many MURs would you complete for patients with asthma/diabetes?

14. What do you consider to be the positive aspects of the current MUR service provided to patients with asthma/diabetes?

*Prompts: Perhaps think of the positive aspects for the patient, e.g. helping patients/carers manage medicines, improved relationship with patients.*

15. What do you consider to be the limitations of the current MUR service provided to patients with asthma/diabetes?

I now want to move on and talk about potentially inappropriate prescribing. Can I ask you to read this definition please?

*[Hand participant a card with the definition printed on it].*

**Potentially inappropriate prescribing (PIP)**

Whilst there is no explicit definition for PIP, it encompasses:

- The over-use of medications at a higher dose/frequency, or for a longer duration than clinically indicated;
- The under-use of medically indicated medications;
- The mis-use of medications that have documented drug-drug interactions or drug-disease interactions.

**Social/professional role and identity**

16. Thinking about potentially inappropriate prescribing in older people, what would you consider your contribution/responsibilities to be as a community pharmacist, in ensuring medicines are prescribed appropriately for older people?

*Prompts: Do you consider it to be your responsibility; are there any aspects beyond your contribution/responsibility; is there anyone else you think is responsible for these aspects beyond your contribution/responsibility?*

**Community pharmacists’ experience of screening tools**

As I mentioned at the very start, I am interested in the use of screening tools.

Following on from MURs, there is currently a lot of interest in screening tools which assess prescribing appropriateness. Can I ask you to read this description below please?

*[Hand participant a card with the definition printed on it].*

**Screening tools**

Screening tools are used to identify instances of potentially inappropriate prescribing. They often consist of lists of drugs, drug-classes or dosages known to cause harmful effects. Such tools are defined as explicit tools, e.g. STOPP/START. STOPP is a prescribing tool which is used to identify over- or mis-prescribing, and START is a prescribing tool used to identify under-prescribing.

An example of a STOPP criterion which identifies over-prescribing would be: *‘The prescription of a proton pump inhibitor (PPI) at maximum dose for more than 8 weeks’;* and an example of a START criterion which identifies under-prescribing would be: *‘The omission of statins in a patient with documented cardiovascular disease (CVD)’*. Some of the criteria can only be used if you have access to clinical information such as diagnosis or clinical test results.

Another type of screening tool is an implicit tool, the utilisation of which requires professional judgement and clinical information, e.g. the Medicines Appropriateness Index (MAI).

An example of a MAI criterion would be: *‘Is there an indication for this drug’*.

I have prepared a clinical scenario in order to demonstrate how a screening tool would work in practice.

*[Give participant a card with the clinical scenario printed on it].*

**Clinical scenario**

Patient initials: AM

Sex: Male

Age: 67 years

| **Repeat Prescriptions** | | | |
| --- | --- | --- | --- |
| **Date issued** | **Drug** | **Directions** | **Quantity** |
| 18/12/2015  18/12/2015  18/12/2015  23/10/2015  23/10/2015  23/10/2015 | Omeprazole 20 mg capsules  Prednisolone GR 2.5 mg tablets  Prednisolone GR 5 mg tablets  Omeprazole 20 mg capsules  Prednisolone GR 2.5 mg tablets  Prednisolone GR 5 mg tablets | Take TWO capsules daily  Take ONE tablet daily to keep symptoms under control  Take ONE tablet daily to keep symptoms under control  Take TWO capsules daily  Take ONE tablet daily to keep symptoms under control  Take ONE tablet daily to keep symptoms under control | 112  56  56  112  56  56 |

In the absence of clinical data, the following STOPP criterion can be applied:

*‘The prescription of a proton pump inhibitor (PPI) at maximum dose for more than 8 weeks’.*

In the absence of clinical data, the following START criterion can be applied:

*‘Bisphosphonates in patients taking maintenance corticosteroid therapy’.*

In the absence of clinical data, the following STOPP criterion cannot be applied:

*‘Long-term corticosteroids (>3 months) as monotherapy for rheumatoid arthritis or osteoarthritis’.*

In the absence of clinical data, the following START criterion cannot be applied:

*‘Disease-modifying anti-rheumatic drug (DMARD) with active moderate-severe rheumatoid disease lasting >12 weeks’.*

17. Prior to your participation in this study, have you encountered the term screening tool, and if so what is your understanding/experience of screening tools?

18. Do you think a screening tool would be useful in conducting MURs in older patients?

***Behavioural Elicitation***

*“At present, the current MUR service is restricted to patients with asthma or diabetes. At this stage it would be helpful if you could think of MURs in more general terms and imagine yourself conducting a MUR for an older patient, not specifically an older patient with asthma/diabetes. The subsequent questions relate to your views on the use of a screening tool to conduct a MUR in an older person. The screening tool would be used to assess the appropriateness of prescribing in all older patients (aged ≥65 years), and would not be specific to those with asthma/diabetes”.*

**Knowledge**

19. As a community pharmacist, what knowledge do you have that would enable you to use a screening tool when conducting MURs in older people, as a way to assess the appropriateness of prescribing in this population group?

*Prompts: Clinical knowledge; specific resources; knowledge of patient’s clinical picture; knowledge of guidelines; preferred guidelines; knowledge/experience of inappropriate prescribing.*

20. What training would help you in using a screening tool to conduct MURs in older people, as a way to assess the appropriateness of prescribing in this population group?

*Prompts: Education on the evidence surrounding inappropriate prescribing and the problems associated with it; education on the different types of screening tools available (including pros and cons of each); provision of specific resources/guidelines.*

**Skills**

21. As a community pharmacist, what skills do you have that would enable you to use a screening tool, as a way to assess the appropriateness of prescribing in this population group?

*Prompts: Perhaps think of: skills that help you engage with patients, carers, other HCPs; think of your skills in problem solving/time management.*

22. Can you think of any specific skills training you feel would help you when using a screening tool to conduct MURs in older people, as a way to assess the appropriateness of prescribing in this population group?

*Prompts: Perhaps training in: screening tool utilisation; additional training in MUR service provision; training in how to address issues of inappropriate prescribing (how to address issues with GP/patient/carer).*

**Beliefs about capabilities**

23. In what situations would you feel confident about using a screening tool to conduct MURs in older people, as a way to assess the appropriateness of prescribing in this population group?

*Prompts: It might be helpful to think of situations in which you feel confident conducting current MURs in patients with asthma/diabetes. For example, when you: have adequate staff/time; know/don’t know the patient personally; have a good relationship with the patient’s GP; are familiar with the patient’s clinical picture.*

24. In what situations would you feel less confident about using a screening tool to conduct MURs in older people, as a way to assess the appropriateness of prescribing in this population group?

*Prompts: Again it might be helpful to think of situations in which you do not feel confident conducting current MURs in patients with asthma/diabetes. For example, when you do not: have adequate staff/time; know the patient personally; have a good relationship with the patient’s GP; know the patient’s clinical picture.*

**Optimism**

25. How optimistic are you that using a screening tool to conduct MURs in older people, would help you assess the appropriateness of prescribing in this population group?

26. In instances where participants are not optimistic, ask why they do not think a screening tool would help them assess the appropriateness of prescribing in this population group.

**Beliefs about consequences**

27. Can you think of any benefits of using a screening tool to conduct MURs in older people, as a way to assess the appropriateness of prescribing in this population group?

*Prompts: For patients, carers, NHS, yourself, short- and long-term consequences.*

28. Can you see any problems associated with using a screening tool to conduct MURs in older people, as a way to assess the appropriateness of prescribing in this population group?

*Prompts: As for question 27.*

**Reinforcement**

29. What would encourage you to use a screening tool to conduct MURs in older people, as a way to assess the appropriateness of prescribing in this population group?

*Prompts: Rewards/incentives for yourself/the pharmacy e.g. services income, personal rewards, professional recognition; evidence of clinical benefit to patient.*

30. What would discourage you from using a screening tool to conduct MURs, as a way to assess the appropriateness of prescribing in this population group?

*Prompts: A lack of: rewards/incentives for yourself/the pharmacy e.g. services income, personal rewards, professional recognition; evidence of clinical benefit to patient.*

**Intentions**

31. If you had access to a screening tool developed for use in the community pharmacy setting, how would you plan to use it as a way to conduct a MUR in an older patient, as a means to assessing the appropriateness of the medicines prescribed for that patient?

32. Are there any circumstances that would prevent you from using such a screening tool?

**Goals**

33. To what extent would it be a priority for you to use a screening tool to conduct MURs in older people, as a way to assess the appropriateness of prescribing in this population group?

*Prompts: Perhaps think of whether you see it as a priority.*

34. In what circumstances would it be less important to use a screening tool to conduct MURs in older people, as a way to assess the appropriateness of prescribing in this population group?

*Prompts: Think of other priorities you have as a community pharmacist.*

**Memory, attention and decision processes**

35. Can you tell me how you would remember to address any issues identified whilst using a screening tool to conduct a MUR in an older patient, as a way to assess the appropriateness of prescribing in that patient?

*Prompts: Perhaps it would be helpful to think of how you currently remember to address such issues in asthmatic or diabetic patients.*

36. Are there any circumstances in which you would forget to address such issues?

*Prompts: Perhaps the work environment; culture within the pharmacy; material/resources available; critical incidents within the pharmacy.*

**Environmental context and resources**

37. What resources/support might help you to use a screening tool to conduct MURs in older people, as a way to assess the appropriateness of prescribing in this population group?

*Prompts: Clinical resources; adequate staffing; rewards for yourself/the pharmacy e.g. services income, personal rewards, professional recognition.*

38. Can you think of any factors in terms of resources/support that might prevent you from using a screening tool to conduct MURs in older people, as a way to assess the appropriateness of prescribing in this population group?

**Social influences**

39. Who would influence your decision to use a screening tool to conduct MURs in older people, as a way to assess the appropriateness of prescribing in this population group?

*Prompts: Patients, carers, GPs, hospital consultant, pharmacy colleagues, other HCPs. Can you tell me more about how you imagine this would happen and what their influence would be?*

**Emotion**

40. How would you feel about using a screening tool to conduct MURs in older people, as a way to assess the appropriateness of prescribing in this population group?

*Prompts: Encouraged by prospect of additional responsibility; intimidated by lack of experience in utilisation of screening tools; disheartened by the lack of resources/funding/recognition.*

**Behavioural regulation**

41. If you detected an issue of inappropriate prescribing whilst using a screening tool to conduct a MUR in an older patient, are there any ways in which you could monitor whether or not it has been resolved?

*Prompts: Perhaps it would be helpful to think of: current practice and how you address issues detected whilst providing MURs in asthmatic and diabetic patients; workplace protocols you have in place.*

42. If the issue was not resolved, are there any strategies you would use in order to overcome this?

*Prompts: As for question 40.*

**Closing the interview**

That brings us to the end of the interview.

In regard to discussing the use of a screening tool to conduct MURs in older people, is there anything else about PIP, MURs, screening/prescribing tools that you feel has not been covered?

Thank you very much for taking the time to speak to me today.

*[Turn off digital recorder]*
